# Perioperative and short-term outcomes of laparoscopic liver resection for recurrent hepatocellular carcinoma: A retrospective study comparing open hepatectomy

**DOI:** 10.3389/fonc.2022.956382

**Published:** 2022-10-17

**Authors:** Dandan Bao, Yiren Hu, Chenghao Zhang, Yibang Jin, Pengwei Wang, Yinfeng Lin, Wei Wang, Yunfeng Shan

**Affiliations:** ^1^ Department of General Surgery, The Third Clinical Institute Affiliated to Wenzhou Medical University, Wenzhou People’s Hospital, Wenzhou, China; ^2^ Department of Emergency Medicine, Zhejiang Chinese Medical University, Wenzhou People’s Hospital, Wenzhou, China; ^3^ Department of Oncology, The Third Clinical Institute Affiliated to Wenzhou Medical University, Wenzhou People’s Hospital, Wenzhou, China; ^4^ The Central Blood Station of Taixing People's Hospital, Jiangsu, China; ^5^ Department of Hepatobiliary and Pancreatic Surgery, The First Affiliated Hospital of Wenzhou Medical University, Wenzhou, China

**Keywords:** recurrent hepatocellular carcinoma, laparoscopic liver resection, open liver resection, propensity score matching, recurrence-free survival

## Abstract

**Background:**

To compare the perioperative and short-term outcomes of laparoscopic liver resection (LLR) and open liver resection (OLR) in recurrent hepatocellular carcinoma (rHCC) based on propensity score matching (PSM) to investigate therapeutic safety, efficacy, and value for clinical application.

**Methods:**

Forty-nine patients with rHCC who underwent surgery at Wenzhou People’s Hospital between January 2017 and March 2022 were retrospectively analyzed and classified into LLR (n=30) and OLR (n=22) cases based on the surgical method. Thirty-eight patients were screened using PSM for data analysis to compare basic clinical characteristics, perioperative outcomes, and postoperative recurrence in both groups.

**Results:**

Before PSM, the tumour diameter was larger, tumor staging (BCLC staging system), intraoperative blood loss, units of blood transfused, constituent ratio of liver cirrhosis, incidence of MVI and intravascular tumour thrombus and postoperative complication were higher, and duration of hospital stay was significantly longer in the OLR group compared to those in the LLR group (*p* < 0.05). After PSM, there were no significant differences regarding tumour diameter, MVI incidence, blood transfusion amount or postoperative complication rate in the LLR and OLR groups. The tumor staging, incidence of vascular cancer thrombus, intraoperative blood loss and postoperative duration of hospitalisation were significantly higher in the OLR group than in the LLR group (*p*<0.05). The difference in recurrence-free survival (RFS) between the two groups was not statistically significant (*p* = 0.383).

**Conclusion:**

LLR for recurrent hepatocellular carcinoma can reduce intraoperative blood loss and postoperative complication rate, shorten the duration of hospitalisation, and is superior to OLR regarding perioperative and short-term efficacy, demonstrating good safety and feasibility.

## Introduction

Hepatocellular carcinoma (HCC) is a common primary malignancy of the liver with a poor prognosis, and is the fifth most common cancer and the second leading cause of cancer-related death worldwide (1). Liver resection is recognized as the most effective method to treat HCC, but the postoperative intrahepatic recurrence rate remains as high as 80%, and therefore the need for repeat liver resection has increased accordingly (2, 3). Conventional open liver resection is a more mature procedure but is traumatic and involves lengthy recovery, particularly for patients of advanced age or with cirrhosis or comorbidities, which can easily lead to various complications, poor prognosis and affect quality of life. In the past decade, conventional open liver resection (OLR) has gradually been replaced by laparoscopic liver resection (LLR) with the development of minimally invasive techniques and improved postoperative management (4). Many studies have indicated no significant difference in long-term prognosis between LLR and OLR, but laparoscopic surgery can significantly reduce the incidence of postoperative complications such as intraoperative blood loss and postoperative pain, as well as shorten the duration of hospitalization (5, 6). However, most previous studies include potential case selection bias. for example, patients with HCC undergoing OLR tend to be older, have larger tumours, and few patients with recurrent HCC (rHCC) underwent evaluation for repeat liver resection. The aim of this propensity score-matched (PSM) study was to investigate the efficacy and safety of LLR by comparing the perioperative outcomes and long-term prognostic results of LLR versus OLR in rHCC.

## Data and methods

### General characteristics

A total of 52 patients with rHCC undergoing liver resection at Wenzhou People’s Hospital between January 2017 and March 2022 were included in the study. These included 30 patients in the LLR group and 22 in the OLR group. Patient data were collected without strict randomisation. To reduce potential selection bias in the study, 38 cases were screened using PSM for preoperative characteristics (sex, age, BMI, total bilirubin, albumin), liver function test results, maximum tumour diameter, and surgical difficulty score; Nineteen cases each in the LLR and OLR groups were included. The study was approved by the hospital Institutional Review Board (approval number: YK-2022-001).

Inclusion criteria: (1) confirmed diagnosis of rHCC; (2) preoperative assessment indicated a Child–Pugh liver function grade A; (3) no other serious concomitant disease, such as cardiac dysfunction grade III or higher, myocardial infarction, or severe liver, lung, kidney, or hematopoietic system diseases; (4) no psychiatric disorders impacting the ability to cooperate with medical personnel; (5) availability of complete clinical data. Exclusion criteria: (1) serious concomitant disease; (2) Child–Pugh liver function grade B or C; (3) patients converted from LLR to OLR; (4) patients with concomitant systemic disease requiring surgical treatment or who underwent other major surgeries within the previous 2 months; (5) patients with missing clinical information.

LLR should follow the safety principle of OLR in the treatment of HCC, the indications and contraindications of Laparoscopy surgery should be consistent with open surgery in principle, patient’s general condition, liver reserve function, tumour size, location and number should be comprehensively evaluated. The indications of LLR for rHCC are as follows: (1) Tumour diameter ≤5cm and located in peripheral liver segment (Couinaud segments II, III, IV, V and VI) is preferred for LLR. (2) Tumours less than 5cm in diameter at difficult locations and tumours 5 to 10cm in diameter can be performed by experienced surgeons. (3) Patients with tumors adjacent to or invading the first or second hilus should be performed at an experienced medical center. The contraindications of LLR include any contraindications of OLR. Due to the particularity of laparoscopy, it also include the following situations: (1) Pneumoperitoneum intolerance patients. (2) Severe abdominal adhesion leads to difficult to expose the tumour. (3) The tumours invade or are adjacent to important structures, leading to the failure of laparoscopic surgery.

### Surgical method

**Figure 1:**
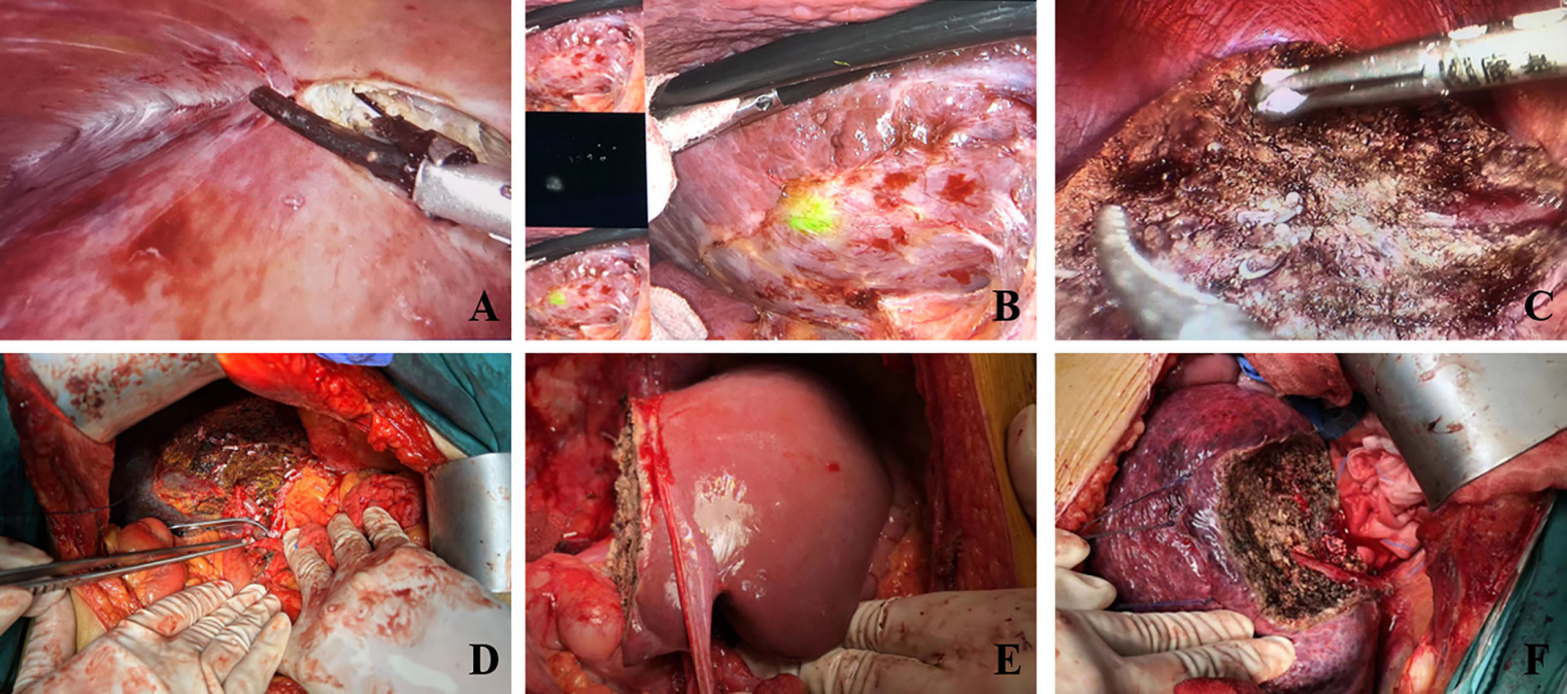
Intraoperative images of each surgical method. **(A)** Laparoscopic adhesiolysis for recurrent HCC. **(B)** Fluorescence imaging of recurrent HCC. **(C)** Laparoscopic left hemihepatectomy. **(D)** Hepatic pedicle blocked using Pringle maneuver. **(E)** Open right hemihepatectomy. **(F)** Open partial hepatectomy.

LLR group: Complete laparoscopic surgery was performed under general anaesthesia with the patient in the supine position and appropriately elevated in the hepatic area. The patient’s legs were separated depending on the site of the tumour (generally in the caudate or right posterior lobes of the liver), and partial or segmental liver resection was performed. A CO2 pneumoperitoneum was established and abdominal pressure was maintained at 12–14 mmHg. A conventional 5-port approach was used, a laparoscopic lens was placed, and a trocar puncture was performed under direct vision with a primary working port size of 1.2 cm, and 0.5 cm for the remaining working ports. An ultrasonic knife was used to separate abdominal adhesions, the surgical field was exposed, and a haemostatic band was placed at the hepatic portal. The location and size of the tumour were confirmed under direct vision or laparoscopic ultrasound guidance, and a pre-resection line was marked 2 cm from the tumour margin or at the anatomical division of the liver segment. The hepatic parenchyma was gradually dissected along this line using the ultrasonic knife. Ducts < 3 mm were directly severed using the ultrasonic knife after coagulation; ducts between 3–7 mm were severed after clipping with Hem-o-lok clips, and ducts > 7 mm were severed using a laparoscopic anastomosis device. The Pringle manoeuvre was used to clamp the hepatic portal and limit blood loss. The tumour was completely resected, placed in a specimen bag, and removed through the widened periumbilical incision. The wound was irrigated, bleeding was stopped and a drainage tube was placed as required; the incisions were sutured, as shown in **Figures 1A–C**.

OLR group: Open liver resection was performed under general anaesthesia. After sterile drapes were placed and the liver was appropriately elevated, an oblique or reverse L-shaped incision about 15–25 cm in length was created under the right rib margin. The perihepatic adhesions were freed, exposing the surgical field, and a haemostatic band was placed at the hepatic portal. The location and size of the tumour were confirmed under direct vision or laparoscopic ultrasound guidance, and a pre-resection line was marked 2 cm from the tumour margin or at the anatomical division of the liver segment. The Pringle manoeuvre was used to clamp the hepatic portal. The hepatic parenchyma was gradually dissected using the ultrasonic knife. Ducts were severed after clipping with Hem-o-lok clips, and the tumour was completely resected and removed, as shown in **Figures 1D–F**.

### Observational indices and evaluation methods

The gender, age, BMI, white blood cell count (WBC), platelet count (PLT), international normalised ratio (INR), alanine transaminase (ALT), aspartate transaminase (AST), alkaline phosphatase (ALP), gamma-glutamyltransferase (GGT), Child–Pugh score, total bilirubin, albumin, HBV infection status, the presence of cirrhosis, tumour grade and MVI, as well as number of tumours, maximum tumour diameter, and vascular tumour thrombus of the patients were recorded. rHCC was diagnosed and evaluated preoperatively using enhanced computed tomography (CT) or enhanced magnetic resonance imaging (MRI) in both groups. Surgical duration, surgical difficulty score (7), intraoperative blood loss, units of blood transfused, postoperative complications, and length of hospital stay were recorded for both groups. Postoperative complications included biliary leakage, ascites, pleural effusion, wound infection, and venous thrombosis. Tumour staging was according to the Barcelona Clinic Liver Cancer (BCLC) classification.

The recurrence rate of HCC was observed over a follow-up period of 6–50 months after surgery in both groups. Recurrence-free survival (RFS) was defined as the length of time between the current surgical treatment and recurrence.

### Statistics

SPSS 25.0 statistical software (IBM Corp., Armonk, NY) was used for data analysis. Measurement data were expressed as (mean ± SD) or median (interquartile ranges), and the independent samples t-test or Mann–Whitney test were used to compare differences between groups. Count data were expressed as n (%), and Pearson chi-square test or Fisher’s exact probability method was used to compare differences between groups. The Kaplan–Meier method was used to assess RFS. P < 0.05 was considered statistically significant.

## Results

### Baseline clinical characteristics of the patient groups before and after PSM

The baseline clinical characteristics of the two groups before and after PSM are summarized in **Table 1**. Before PSM, maximum tumour diameter, tumor staging (BCLC staging system), constituent ratio of liver cirrhosis, and the incidence of MVI and intravascular tumour thrombus exhibited statistically significant differences between the two groups (p = 0.005). Patients who underwent OLR tended to have a higher proportion of previous surgeries of open hepatectomy than those who underwent laparoscopic hepatectomy (63.6% vs. 36.4%). However, patients who underwent LLR had a lower rate of previous open hepatectomy than those who underwent laparoscopic hepatectomy (43.4% vs. 56.7%). After PSM, the baseline clinical characteristics between the LLR and OLR groups were well balanced. The tumor staging and intravascular tumour thrombus were significantly higher in the OLR group compared to those in the LLR group, while there were no statistically significant between-group differences in other baseline clinical characteristics.

**Table 1:** Baseline preoperative clinical characteristics of the two patient groups before and after PSM.

Indicator		Before PSM				After PSM			
		LLR group (n = 30)	OLR group (n = 22)	test value	*p*-value	LLR group (n = 19)	OLR group (n = 19)	test value	*p*-value
Gender [Male, n (%)]	26 (86.7)		19 (86.4)	x^2 =^ 0.001	0.975	17 (89.5)	16 (84.2)	x^2 =^ 0.230	0.631
Age (years)	59.6 ± 11.9		60.1 ± 10.6	t=0.193	0.848	61.6 ± 12.9	59.2 ± 9.9	t=0.663	0.512
BMI (kg/m^2^)	23.2 (22.5-24.6)		24.1 (22.5-24.9)	Z=-0.889	0.374	23.2 (21.2-23.7)	23.9 (22.6-24.9)	Z=-1.007	0.314
WBC (10^9^/L)	5.2 ± 1.8		4.9 ± 1.7	t=0.727	0.470	5.2 ± 1.8	4.9 ± 1.8	t=0.580	0.566
HGB (g/L)	135.4 ± 15.4		134.6 ± 20.6	t=0.160	0.874	133.0 ± 16.4	132.6 ± 19.5	t=0.063	0.950
PLT (10^9^/L)	166.0 ± 73.7		151.3 ± 70.7	t=0.724	0.472	167.2 ± 76.8	153.5 ± 75.7	t=0.553	0.583
INR	1.1 ± 0.1		1.1 ± 0.1	t=0.913	0.366	1.1 ± 0.1	1.1 ± 0.1	t=1.508.	0.141
ALT (U/L)	20.1 (16.0-28.8)		24.8 (19.8-34.5)	Z=-1.781	0.075	20.0 (14.0-34.0)	25.0 (20.0-36.0)	Z=-1.827	0.128
AST (U/L)	23.7 (20.8-30.3)		28.3 (22.8-39.0)	Z=-1.550	0.121	23.0 (20.0-31.0)	28.0 (23.0-38.0)	Z=-1.522	0.128
ALP (U/L)	75.3 (55.5-106.3)		96.0 (74.3-142.3)	Z=-1.585	0.110	73.0 (54.0-110.0)	112.0 (66.0-143.0)	Z=-1.439	0.105
GGT (U/L)	34.0 (21.0-79.8)		40.0 (27.8-152.3)	Z=-1.260	0.208	34.0 (21.0-77.0)	36.0 (27.0-150.0)	Z=-1.081	0.290
Child–Pugh score	5.2 (5.0-5.2)		5.2 (5.0-5.3)	Z=-0.474	0.636	5.0 (5.0-5.0)	5.0 (5.0-5.0)	Z=-0.413	0.680
Total bilirubin (μmol/L)	14.9 ± 6.9		14.6 ± 6.2	t=0.191	0.849	13.9 ± 6.1	14.7 ± 6.6	t=0.410	0.684
Albumin (g/dL)	41.9 ± 6.7		40.0 ± 6.0	t=1.025	0.310	42.4 ± 7.0	39.8 ± 6.2	t=1.204	0.237
Number of tumours	1.3 (1–2)		1.1 (1-1.1)	Z=-1.405	0.160	1.0 (1.0-1.0)	1.0 (1.0-1.0)	Z=-0.453	0.651
Maximum tumour diameter (cm)	2.0 (1.5-2.6)		4.0 (2.2-10.0)	Z=**-3.086**	**0.002**	2.0 (1.5-4.0)	3.0 (2.0-7.5)	Z=-0.829	0.413
AFP	3.9 (2.2-10.5)		3.5 (2.1-94.0)	Z=-0.019	0.985	3.7 (2.8-8.1)	3.4 (2.1-110.2)	Z=0.001	1.000
HBV	24 (80.0)		17 (77.3)	x^2 =^ 0.057	0.812	13 (68.4)	14 (73.7)	x^2 =^ 0.128	0.721
Liver cirrhosis	13 (43.3)		16 (72.7)	x^2 =^ **4.446**	**0.035**	11 (57.9)	14 (73.7)	x^2 =^ 1756	0.179
BCLC stage (A/B and C)	24 (80.0)/6 (20.0)		7 (31.8)/15 (68.2)	**x^2 =^ 12.239**	**0.001**	14 (73.7)/5 (26.3)	7 (36.8)/12 (63.2)	**x^2 =^ 5216**	**.0.022**
MVI (0/1 and 2)	25 (83.3)/5 (16.7)		11 (50.0)/11 (50)	x^2 =^ **6.620**	**0.010**	15 (78.9)/4 (21.1)	10 (52.6)/9 (47.4)	**x^2 =^ 2.923**	**0.087**
Intravascular tumour thrombus [n (%)]	1 (3.3)		8 (36.4)	x^2 =^ **9.675**	**0.006**	0 (0)	6 (31.6)	**x^2 =^ 4.948**	**0.026**
Previous hepatectomy (Open/laparoscopic)	13 (43.3)/17 (56.7)		14 (63.6)/8 (36.4)	x^2 =^ 2.096	0.148	9 (47.4)/10 (52.6)	12 (63.2)/7 (36.8)	x^2 =^ 0.958	0.328

LLR, laparoscopic liver resection; OLR, laparoscopic liver resection; BMI, body mass index; HGB, hemoglobin; PLT, Platelets; INR, international Normalized Ratio; ALT, alanine transaminase; AST, aspartate transaminase; ALP, alkaline phosphatase; GGT, gamma-glutamyl transpeptidase. The bold values represent statistically significant P values (P< 0.05). Data represent mean ± standard deviation/median (interquartile ranges) or number of patients.

### Conditions of the two patient groups during the perioperative and follow-up periods before and after PSM

The conditions of patients in both groups during the perioperative period before and after PSM are presented in **Table 2**. Before PSM, intraoperative blood loss and amount of blood transfusion were significantly lower in the LLR group (p < 0.05), and duration of hospital stay. After PSM, the intraoperative blood loss and duration of postoperative hospital stay was significantly less (p < 0.05) in the LLR group compared to that in the OLR group.

**Table 2:** Conditions of the two patient groups during the perioperative and follow-up periods before and after PSM.

Indicator	Before PSM				After PSM			
	LLR group (n=30)	OLR group (n=22)	z value	*p*-value	LLR group (n=19)	OLR group (n=19)	z value	*p*-value
Surgical difficulty score	6.6 (4.0-8.0)	6.3 (4.0-7.3)	-0.684	0.494	6.0 (4.0-8.0)	6.0 (4.0-8.0)	-0.389	0.697
Surgical duration (min)	189.5 (147.5-254.0)	225.0 (190/0-277.5)	-1.538	0.124	184.0 (150.0-292.0)	220.0 (190.0-270.0)	-1.183	0.237
Intraoperative blood loss (ml)	100.0 (50.0-200.0)	350.0 (20.0-500.0)	**-4.341**	**<0.001**	200.0 (50.0-200.0)	300.0 (200.0-500.0)	**-2.606**	**0.009**
Amount of blood transfusion	0 (0-0)	0 (0-255.0)	**-2.847**	**0.004**	0 (0-0)	0 (0-0)	-1.466	0.143
Negative margin (cm)	1.5 (1.0-1.6)	1.0 (1.0-1.5)	-0.880	0.379	1.5 (1.0-2.0)	1.0 (1.0-1.5)	-0.878	0.380
Duration of hospital stay (day)	13.0 (10.0-15.3)	17.0 (12.8-21.5)	**-2.786**	**0.047**	15.0 (11.0-17.0)	19.0 (15.0-22.0)	**-2.371**	**0.018**
Postoperative RFS (month)	7.0 (4.0-16.0)	11.0 (5.0-22.5)	-1.244	0.214	7.0 (4.0-16.0)	11.0 (5.0-16.0)	-0.865	0.387
Follow-up period (month)	14.0 (8.5-21.3)	21.0 (14.3-30.8)	-1.794	0.079	13.0 (7.0-21.0)	18.0 (12.0-27.0)	-2.031	**0. 042**

Bold value means statistically significant (P<0.05).

Postoperative complications were shown in **Table 3**. Before PSM, the total incidence of postoperative complications including biliary leakage, ascites, pleural effusion, wound infection and venous thrombosis in the LLR group before PSM was lower than that in the OLR group (P < 0.05), and the incidence of ascites in the LLR group was significantly lower than that in the OLR group (< 0.05). After PSM, the incidence of ascites was still significantly lower in LLR group than that in OLR group. There was no significant difference in other postoperative complications between the two groups after PSM (P > 0.05).

**Table 3:** Comparison of postoperative complications between the two groups before and after PSM.

Indicator	Before PSM				After PSM				
	LLR group (n = 30)	OLR group (n = 22)	x^2^ value	*p*-value	LLR group (n = 19)	OLR group (n = 19)	x^2^ value	*p*-value	
Biliary leakage[n (%)]	2 (6.7)	1 (4.5)	0.105	0.745	2 (10.5)	1 (5.3)	0.362	0.544	
Ascites[n (%)]	4 (13.3)	9 (40.9)	**5.147**	**0.023**	1 (5.3)	7 (36.8)	**5.700**	**0.047**	
Pleural effusion[n (%)]	9 (30.0)	10 (45.5)	1.307	0.253	4 (21.1))	8 (42.1)	1.949	0.163	
Wound infection[n (%)]	1 (3.3)	0 (0)	0.748	0.577	1 (5.3)	0 (0)	1.027	0.311	
Venous thrombosis[n (%)]	1 (3.3)	0 (0)	0.748	0.577	1 (5.88)	0 (0)	1.027	0.311	
Total complication rate [n (%)]	17 (56.7)	20 (90.9)	**7.251**	**0.007**	8 (42.1)	12 (63.2)	1.689	0.194	

Bold value means statistically significant (P<0.05).

### Postoperative RFS of the two patient groups before and after PSM

**Figure 2:**
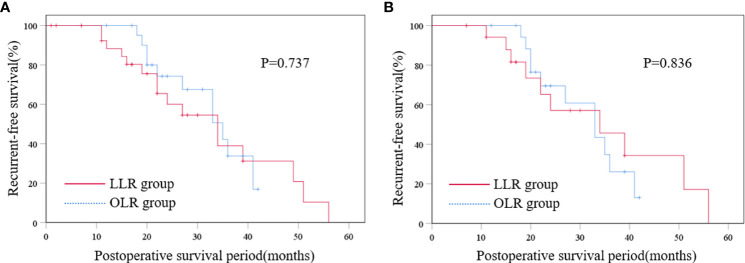
Disease-free survival curve of the two groups before PSM **(A)** and Disease-free survival curve of the two groups after PSM **(B)**. LLR laparoscopic liver resection, OLR open liver resection, PSM propensity score matching.

Before PSM, the median follow-up period was 14.0 months, and the median disease-free survival time was 7.0 months in the LLR group, while the median follow-up period of the OLR group was 21.0 months, and the median disease-free survival time was 11.0 months. After PSM, the median follow-up period was 13.0 months and the median disease-free survival time was 7.0 months in the LLR group, while the median follow-up period was 18.0 months and the median disease-free survival time was 11.0 months in the OLR group. There was no significant difference in the disease-free survival time between the two groups before and after PSM (P > 0.05). The disease-free survival curves of the two groups were shown in **Figure 2**, and there were no statistical differences in RFS between the two groups before and after PSM (P > 0.05).

## Discussion

Primary HCC is one of the most common gastrointestinal malignancies in clinical practice and has a high mortality rate. Liver resection is a common and effective treatment for HCC (8). Tumour recurrence after liver resection remains a major factor influencing the long-term prognosis of patients with HCC, and liver resection for rHCC is associated with more technical challenges compared to primary HCC, including the presence of cirrhosis and abdominal adhesions. Liver resection for rHCC has a higher risk of haemorrhage and intestinal injury that is further complicated by anatomical distortion of the liver due to prior hepatic atrophy/hypertrophy (9). The therapeutic efficacy of conventional open surgery is widely accepted, but it causes more injury to the patient and leads to more complications and slower postoperative recovery. With advances in surgical instrumentation, techniques, and overall understanding of liver anatomy, laparoscopic surgery has been widely used in all fields of surgery and its application in the treatment of malignant tumours has increased rapidly.

Laparoscopic hepatectomy (LLR) has attracted wide attention due to its advantages of less surgical trauma, less postoperative pain and fast recovery. At present, a large number of studies have shown that LLR has significant advantages in the treatment of primary liver cancer, such as reducing blood loss, relieving pain, reducing postoperative complications and shortening hospital stay. The long-term prognosis was similar to or better than that of open hepatectomy (OLR) (10–17). However, only a few studies have been conducted on recurrent HCC (15–18). Therefore, the clinical value and long-term prognosis of LLR in recurrent HCC are still controversial.

In the present study, the perioperative and short-term therapeutic outcomes of LLR and OLR were compared for patients with rHCC using PSM to reduce potential case selection bias and to evaluate its clinical application. The study found that the intraoperative blood loss, amount of blood transfusion and the duration of hospital stay were significantly lower in the LLR group than that in the OLR group (P< 0.05), indicating that LLR group has better perioperative efficacy than OLR group. Postoperative complications of liver resection have a great influence on patients’ condition and prognosis. In the whole sample data, the total incidence of complications in the LLR group was significantly lower than that in the OLR group, indicating that LLR can reduce damage to surrounding tissues and reduce the incidence of postoperative complications, which facilitates early postoperative recovery. There was no statistically significant difference in surgical duration between the two groups, indicating that the relative maturity of laparoscopic techniques and procedures did not increase the incidence of complications due to longer surgical duration. The lack of difference in negative margins of resected specimens suggests that similar surgical outcomes can be achieved when there are no differences in tumour size and surgical difficulty scores between the two groups, and that ensuring negative margins is a major factor influencing postoperative recurrence (19). In addition, we observed no significant difference in recurrence-free survival (RFS) between the two groups, indicating that a similar prognosis can be achieved with both surgical approaches under similar surgical conditions.

The present study possesses some limitations. First, this was a retrospective, non-randomised study, and although PSM was used to eliminate bias in baseline differences, the limitations of PSM itself cannot be ignored. Second, there is a certain selection bias in the surgical resection criteria of patients. The tumour staging in OLR group was significantly higher than that in LLR group before and after PSM, suggesting that tumor resection in LLR group is relatively easy. In the future, larger samples or multicenter studies are needed to reduce the bias and further verify the feasibility and safety of LLR in the treatment of rHCC.

In conclusion, compared to conventional open liver resection, laparoscopic liver resection for recurrent HCC can reduce intraoperative blood loss and blood transfusion requirements, shorten hospital stay, and decrease the rate of postoperative complications; both procedures can achieve a similar long-term prognosis. Therefore, we believe that LLR is a safe and feasible alternative to OLR for rHCC at centres experienced in laparoscopic and liver surgery.

## Data availability statement

The datasets presented in this study can be found in online repositories. The names of the repository/repositories and accession number(s) can be found in the article/**Supplementary Material**

## Author contributions

DB and HY designed the study and wrote the manuscript with contributions from all authors. CZ and YJ collected clinical data. PW and YL analyzed the data. YS provided advice for the project and reviewed the manuscript. WW provided help in data re-collection, statistical analysis and data proofreading during revision process. YS supervised the project. All authors read and approved the final version of the paper.

## Funding

The authors are thankful to the National Nature Science Foundation of China (Grant NO. 81900566). The sponsors have no role in study design, in the collection, analysis and interpretation of data, in the writing of the report, and in the decision to submit the article for publication.

## Conflict of interest

The authors declare that the research was conducted in the absence of any commercial or financial relationships that could be construed as a potential conflict of interest.

## Publisher’s note

All claims expressed in this article are solely those of the authors and do not necessarily represent those of their affiliated organizations, or those of the publisher, the editors and the reviewers. Any product that may be evaluated in this article, or claim that may be made by its manufacturer, is not guaranteed or endorsed by the publisher.
